# Microarray analysis of long non-coding RNA expression profiles in low high-density lipoprotein cholesterol disease

**DOI:** 10.1186/s12944-020-01348-x

**Published:** 2020-07-28

**Authors:** Xinping Wang, Shuxia Guo, Yunhua Hu, Heng Guo, Xianghui Zhang, Yizhong Yan, Jiaolong Ma, Yu Li, Haixia Wang, Jia He, Rulin Ma

**Affiliations:** 1 Department of Public Health, Shihezi University School of Medicine, Shihezi, China; 2 Key Laboratory of Xinjiang Endemic and Ethnic Diseases of the Ministry of Education, Shihezi University School of Medicine, Shihezi, China

**Keywords:** Low HDL-C disease, Long non-coding RNA, Microarray analysis, Platelet activation, TBXA2R, ITGB3, Gene chip

## Abstract

**Background:**

Low high-density lipoprotein cholesterol (HDL-C) disease with unknown etiology has a high prevalence in the Xinjiang Kazak population. In this study, long noncoding RNAs (lncRNAs) that might play a role in low HDL-C disease were identified.

**Methods:**

Plasma samples from 10 eligible individuals with low HDL disease and 10 individuals with normal HDL-C levels were collected. The lncRNA profiles for 20 Xinjiang Kazak individuals were measured using microarray analysis.

**Results:**

Differentially expressed lncRNAs and mRNAs with fold-change values not less than 1.5 and FDR-adjusted *P*-values less than 0.05 were screened. Bioinformatic analyses, including Gene Ontology (GO), Kyoto Encyclopedia of Genes and Genomes (KEGG), and network analyses, were used to determine relevant signaling pathways and predict potential target genes. In total, 381 lncRNAs and 370 mRNAs were differentially expressed based on microarray analysis. Compared with those in healthy individuals, several lncRNAs were upregulated or downregulated in patients with low HDL-C disease, among which TCONS_00006679 was most significantly upregulated and TCONS_00011823 was most significantly downregulated. GO and KEGG pathway analyses as well as co-expression networks of lncRNAs and mRNAs revealed that the platelet activation pathway and cardiovascular disease were associated with low HDL-C disease.

**Conclusions:**

Potential target genes integrin beta-3 (*ITGB3*) and thromboxane A2 receptor (*TBXA2R*) were regulated by the lncRNAs AP001033.3–201 and AC068234.2–202, respectively. Both genes were associated with cardiovascular disease and were involved in the platelet activation pathway. AP001033.3–201 and AC068234.2–202 were associated with low HDL-C disease and could play a role in platelet activation in cardiovascular disease. These results reveal the potential etiology of dyslipidemia in the Xinjiang Kazakh population and lay the foundation for further validation using large sample sizes.

## Background

The dyslipidemia epidemic is a public health concern affecting millions of individuals worldwide. Low high-density lipoprotein cholesterol (HDL-C) disease is an important type of dyslipidemia, as demonstrated by an epidemiological study showing that HDL-C levels are inversely associated with the development of cardiovascular disease [1].

HDL-C is thought to provide protection against cardiovascular disease by regulating cholesterol efflux from peripheral tissues [2], and low HDL-C disease has been recognized as a complex, multifactorial polygenic trait regulated by multiple genes and environmental factors [3].

A previous study has shown that serum HDL-C levels have a strong genetic basis with up to 80% heritability, and detected variants explain only 10% of HDL-C variation [4]. Cholesterol ester transfer protein (*CETP*) [5, 6], ATP-binding cassette transporter A1 (*ABCA1*) [7], apolipoprotein A-1 (*APOA-1*) [8], and cholesterol acyltransferase (*LCAT*) [9] are associated with low HDL-C disease.

In a genome-wide association study, almost all screened single nucleotide polymorphisms (SNPs) were located in non-coding regions; however, the contribution of long non-coding RNAs (lncRNAs) to low HDL-C disease remains unexplored.

LncRNAs, transcripts longer than 200 nucleotides lacking coding potential [10], play crucial roles in various key biological processes and human diseases [11]. However, research on the role of lncRNAs in the development of dyslipidemia is still in its preliminary stage [12, 13].

In the present study, gene chip technology was applied to identify genome-wide differences in the lncRNA composition between the serum of healthy subjects and individuals with low HDL-C disease from a Kazakh population. Differentially expressed lncRNAs and mRNAs were examined and the relationships among these differentially expressed genes and their contributions to low HDL-C disease were analyzed.

## Methods

### Study population

A total of 20 subjects were recruited, including 10 subjects with low HDL-C disease and 10 healthy individuals. The inclusion criterion for subjects with low HDL-C disease was serum HDL-C level < 1.04 mmol/L [14].

Exclusion criteria were the presence of obesity, tumor, diabetes, coronary artery disease (CAD), stroke, and medication history, such as NSAIDs (such as aspirin).

Whole blood (5 mL) from each participant was collected in an EDTA-containing tube and stored at − 80 °C until use.

### Microarray experiment

#### Fabrication of the DNA microarray

The Capital Biotech Human lncRNA+mRNA Array V4.0 was designed with four identical arrays per slide (4 × 180 K format), each array containing probes for approximately 41,000 human lncRNAs and approximately 34,000 human mRNAs. The target lncRNA and mRNA sequences were merged from multiple databases, including 23,898 from GENCODE/ENSEMBL, 14353 from Human LincRNA Catalog [15], 7760 from RefSeq, 5627 from the UCSC database, 13,701 from NRED (ncRNA Expression Database), 21,488 from LNCipedia, 1038 from H-InvDB, 1053 from the Antisense ncRNA pipeline, 407 Hox ncRNAs, 962 UCRs, and 848 from the Chen Ruisheng lab (Institute of Biophysics, Chinese Academy of Science, China). Probes for each RNA were included in duplicate. The array also contained 4974 Agilent control probes.

#### RNA extraction, labeling, and hybridization

Total RNA (including lncRNAs) was extracted from the peripheral serum using TRIzol reagent (Invitrogen, Carlsbad, CA, USA) and purified using the NucleoSpin® RNA Purification Kit (Life Technologies Corporation, Grand Island, NY, USA) according to the manufacturer’s protocol. Spectrophotometry (NanoDrop ND-1000) was performed to determine RNA purity and concentration at OD260/280. RNA integrity was confirmed using 1% formaldehyde denaturing gel electrophoresis (Agilent Technologies, Santa Clara, CA, USA).

#### RNA amplification, labeling, and hybridization

**Fig. 1:**
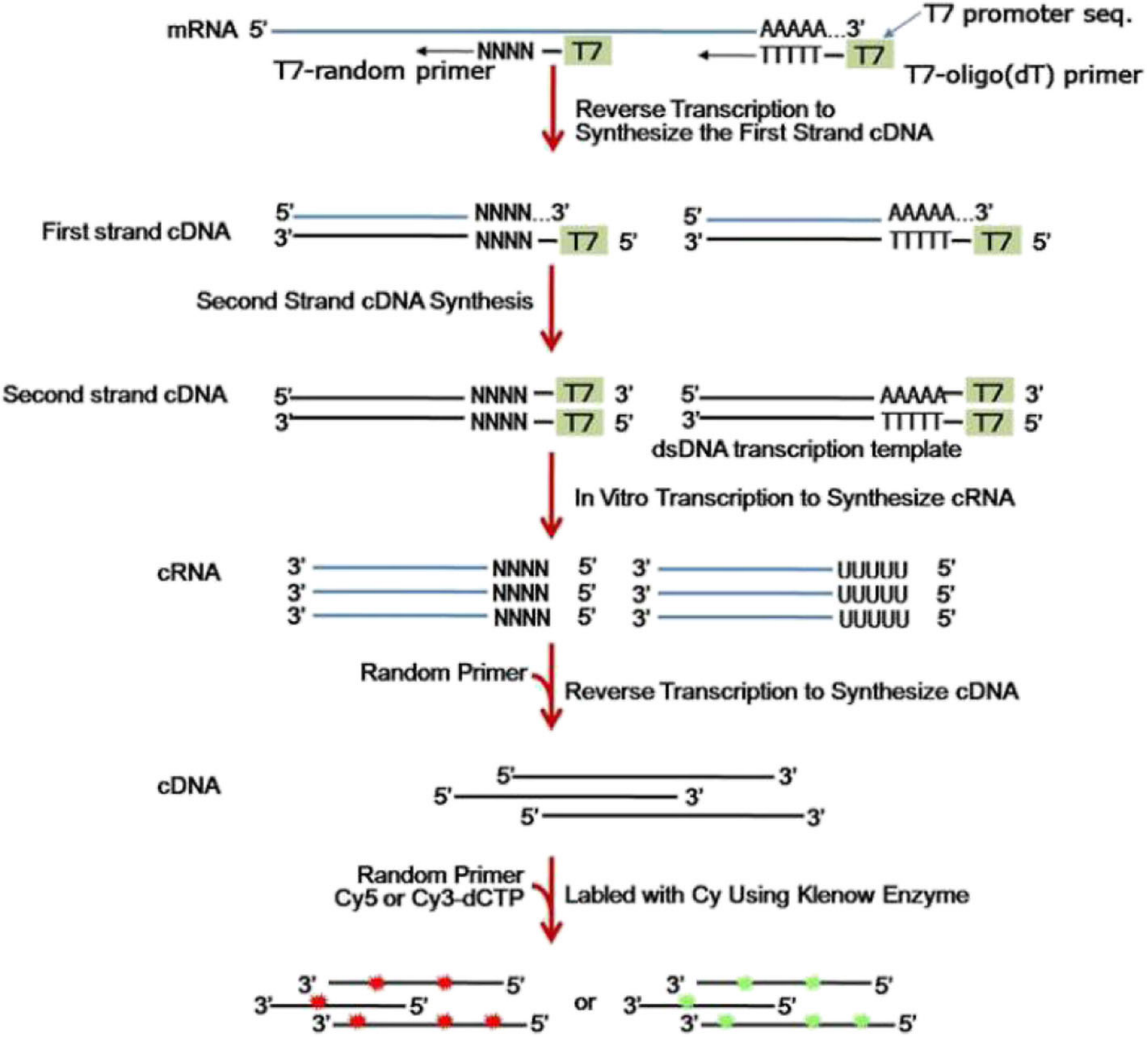
Summary of the cRNA amplification and labeling procedure

The cDNA labeled with a fluorescent dye (Cy5 or Cy3-dCTP) was generated using Eberwine’s linear RNA amplification method and subsequent enzymatic reaction, as described previously [16]. The CapitalBio cRNA Amplification and Labelling Kit (CapitalBio, Beijing, China) was used to produce higher yields of labeled cDNA. The cRNA amplification and labeling procedure is depicted in Fig. 1.

### Microarray imaging and data analysis

Gene Spring GX was used to analyze the lncRNA and mRNA array data, including data aggregation, normalization, and quality control. To select differentially expressed genes, fold changes of ≥1.5 and ≤ 1.5 a *P-*value of < 0.05 for *t*-tests were selected as thresholds. The Adjust Data function of Cluster 3.0 was used to perform Log2 conversion of the data, median positioning centered on genes, and hierarchical clustering with average links for further analysis. Finally, Java TreeView (Stanford University School of Medicine, Stanford, CA, USA) was used to visualize the tree.

### Construction of the coding–non-coding gene co-expression network

Based on a correlation analysis of the differentially expressed lncRNAs and mRNAs, a co-expression network (CNC network) was constructed. For each pair of genes, Pearson correlation coefficients (PCCs) were calculated and pairs with significant correlations were used to construct the network. The open source bioinformatics software Cytoscape was used to generate the lncRNA and mRNA mapping network with a PCC with an absolute value of not less than 0.90 and *P* < 0.05.

### Cis-lncRNA prediction

Cis-acting lncRNA prediction was conducted based on close correlations with a group of expressed protein-coding genes (PCC > 0.90). Each protein-coding gene and lncRNA gene were within 10 kb of each other throughout the genome [17].

### Trans-lncRNA prediction

Blat tools (Standalone BLAT v. 35 × 1 fast sequence search command line tool, downloaded from: http://hgdownload.cse.ucsc.edu/admin/exe/) were used to perform reverse transcription to compare the complete sequence of lncRNA and the 3’UTR of the corresponding co-expressed mRNA with default parameter settings.

### Functional analysis of differentially expressed genes

#### GO and KEGG pathway analyses

The differentially expressed genes were input into KOBAS for annotation and visualization. GO was used to identify the molecular functions overrepresented in the gene profile and KEGG was used to analyze the pathways related to the genes, the significance of GO term enrichment in the differentially expressed mRNAs was denoted by *P*-value (*P* < 0.05 was considered statistically significant).

To study the enrichment of potential genes and gene products in biological processes (BP), cellular components (CC), and molecular functions (MF), a GO analysis of differentially expressed mRNAs was performed. The *P*-value was used to determine significant changes in differentially expressed genes and GO.

To understand whether the dysregulated lncRNAs are involved in the regulation of genes and related signaling pathways associated with low HDL-C disease, potential targets of lncRNAs in the database via cis- and trans-regulation were predicted.

### Statistical analysis

Statistical analyses were performed to compare the two groups in the microarray. Fold change values and Student’s *t*-tests were used to analyze the statistical significance of the microarray results. The threshold value for screening differentially expressed lncRNAs and mRNAs was a fold change of ≥1.5 (*P* < 0.05). The false discovery rate (FDR) was used to correct the *P*-value. The differentially expressed genes were analyzed using Student’s *t*-tests implemented in SPSS (version 22.0; SPSS, Chicago, IL, USA). Fisher’s exact test was used for the KEGG pathway analysis and to compare healthy subjects with patients, where appropriate. PCC was used for the GO analysis and for the prediction of correlations between lncRNAs and protein-coding genes. *P* < 0.05 was considered statistically significant.

## Results

### General characteristics of patients and healthy individuals

Table 1 summarizes the characteristics of all individuals. There were significant differences in HDL-C levels between the two groups (*P* < 0.05). No differences in sex, age, TG, LDL-C, systolic pressure, diastolic pressure, and BMI were observed between the two groups (*P* > 0.05).

**Table 1:** General characteristics of the two groups

Variable	Case	Control	***P***-value
Male/female	5/5	5/5	1
Age (years)	29.540 ± 5.627	29.340 ± 6.707	1
HDL-C, mmol/L	0.725 ± 0.047	1.562 ± 0.117	< 0.001
TG, mmol/L	0.945 ± 0.417	0.821 ± 0.327	0.579
LDL-C, mmol/L	2.170 ± 0.774	1.546 ± 0.502	0.052
Systolic pressure, mmHg	121.250 ± 8.638	125.200 ± 6.210	0.19
diastolic pressure, mmHg	72.050 ± 4.425	73.400 ± 7.321	0.436
BMI, kg/m^2^	20.490 ± 2.676	22.400 ± 1.146	0.089

Values were presented as mean ± SD, t-test or Wilcoxon rank sum test was used to obtained the *P* value for continuous variables, *P* < 0.05 significant

**Fig. 2:**
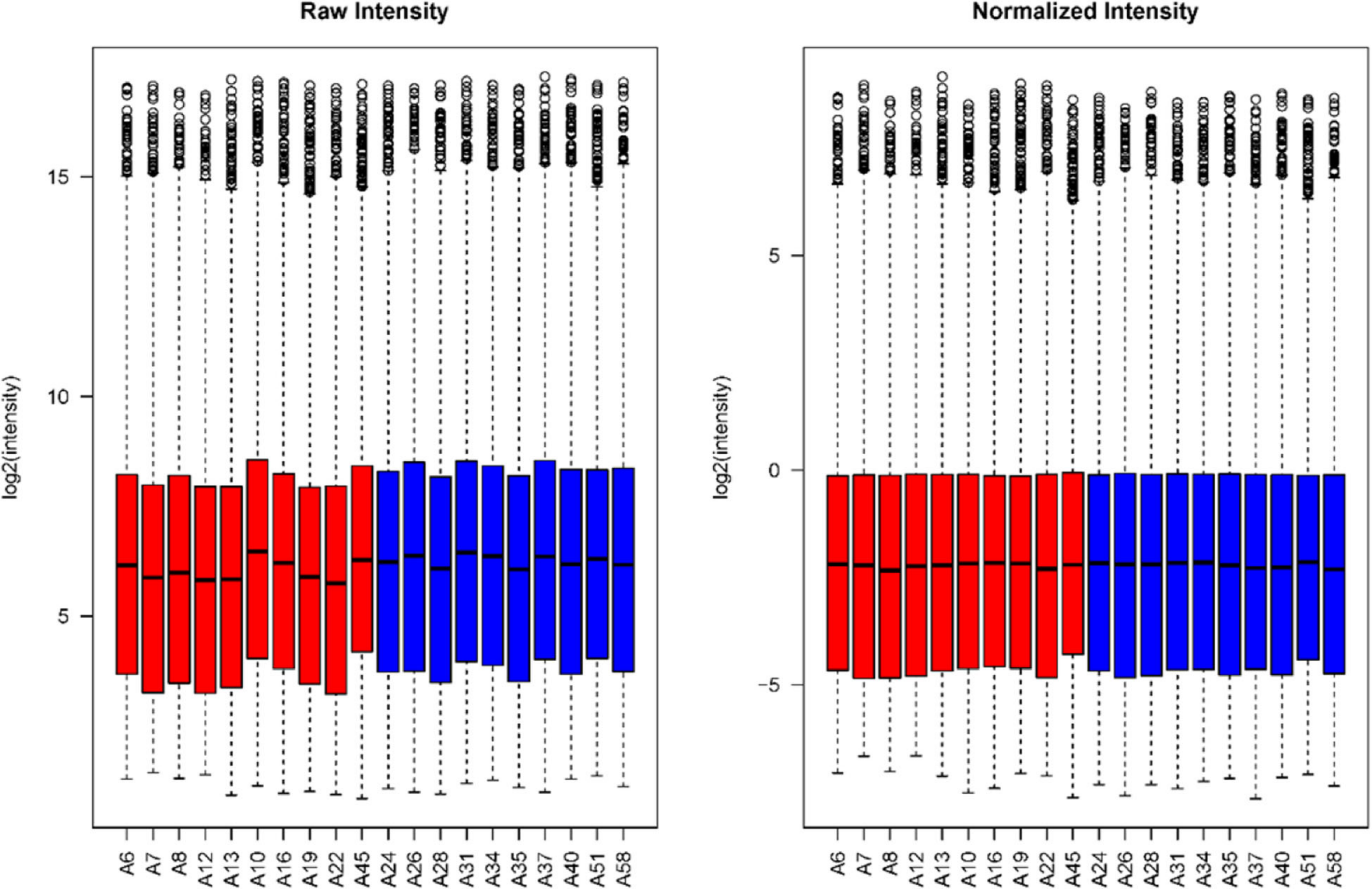
Box plots summarizing the lncRNA and mRNA profiles. After normalization, the distributions of the log2-ratios of fluorescence signals in the tested samples were nearly identical among samples. Blue indicates control samples and red indicates case samples

Total RNA was extracted and purified from the blood samples. The samples were of good quality and could be used for the microarray analysis. The distributions of lncRNA and mRNA levels are shown in Fig. 2.

### lncRNA and mRNA expression profiles

#### Differentially expressed lncRNAs

**Fig. 3:**
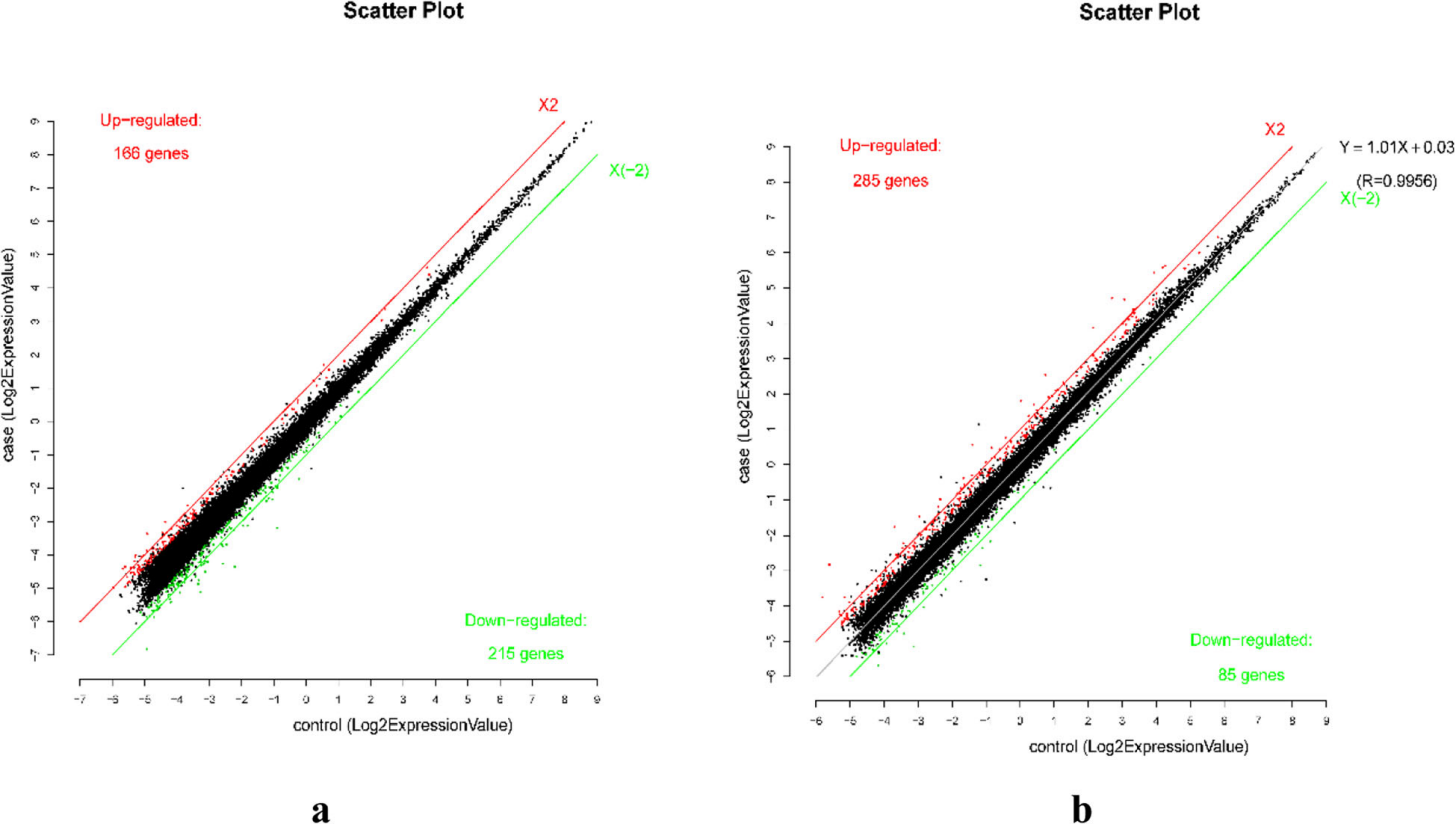
lncRNA (**a**) and mRNA (**b**) expression in the low HDL-C disease group and healthy individuals

**Fig. 4:**
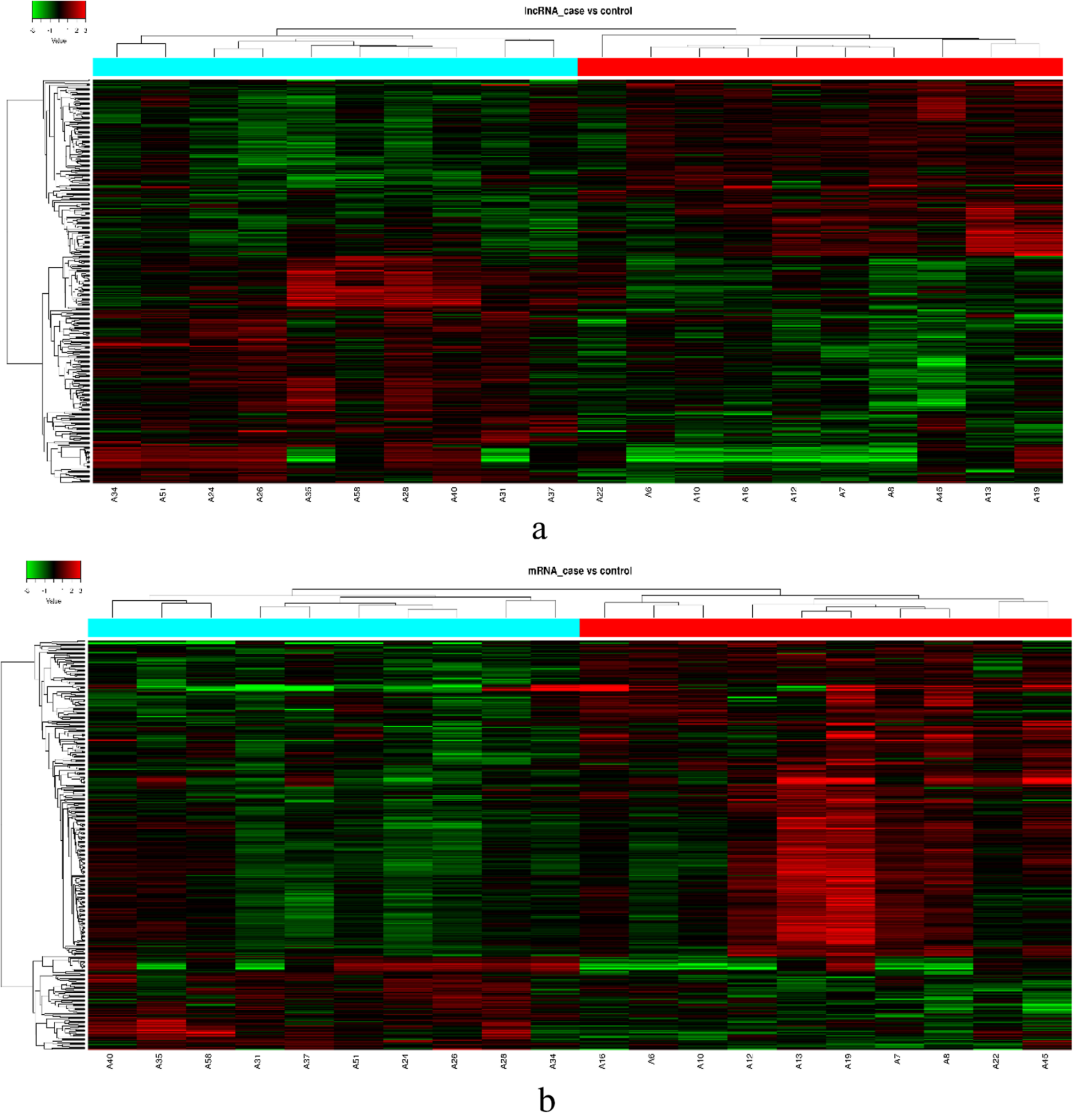
Heat map and hierarchical clustering results for lncRNA and mRNA profiles. **a** lncRNA **b** mRNA. The right shows the group with low HDL-C disease and the left shows normal samples

**Fig. 5:**
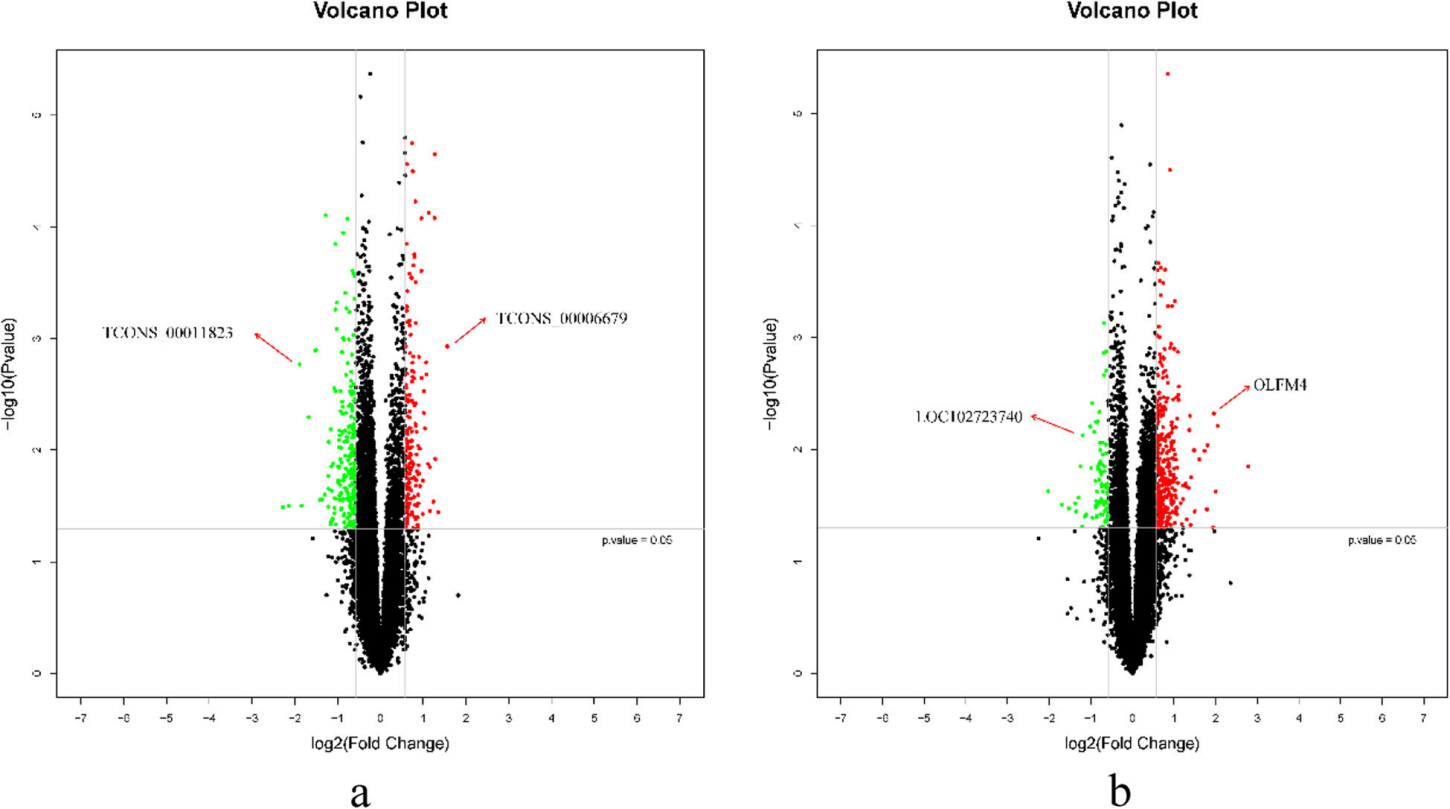
Volcano plot analysis of lncRNA microarray data for differentially expressed lncRNAs (**a**) and mRNAs (**b**) between the two groups

Based on the microarray data, 381 lncRNAs were differentially expressed (fold change ≥1.5, *P* < 0.05) between individuals with low HDL-C disease and healthy individuals, including 166 up-regulated lncRNAs and 215 down-regulated lncRNAs in the low HDL-C disease group (Fig. 3). A hierarchical clustering analysis was used to arrange specimens into groups according to expression levels (Fig. 4). The top 15 differentially expressed lncRNAs are listed in Tables 2 and 3; TCONS_00006679 was the most significantly up-regulated lncRNA (fold change: 2.9543) and TCONS_00011823 was the most significantly down-regulated lncRNA (fold change: 4.8541) (Fig. 5).

**Table 2:** Top 15 up-regulated lncRNAs in individuals with low HDL-C disease compared with those in controls

lncRNA ID	FC	***P***-value	Chromosome	Length (bp)
TCONS_00006679	2.95413	0.0012	3	264
ENST00000531609.1	2.55680	0.0357	14	1442
ENST00000568189.1	2.42925	0.0120	2	735
NR_027309.2	2.40050	0.0001	1	1913
TCONS_00024405	2.39786	0.0000	16	221
TCONS_00012221	2.36027	0.0290	6	240
ENST00000447519.1	2.23095	0.0143	11	1426
NR_073063.1	2.22508	0.0350	6	2057
TCONS_00029160	2.18704	0.0001	21	422
ENST00000592716.1	2.16546	0.0133	19	574
XR_427434.1	2.10105	0.0021	3	605
uc.353+	2.09404	0.0016	13	323
TCONS_00027123	2.05533	0.0064	19	1305
ENST00000454187.1	2.02073	0.0047	9	266
TCONS_00009834	2.02024	0.0030	5	1274

**Table 3:** Top 15 down-regulated lncRNAs in individuals with low HDL-C disease compared with those in controls

lncRNA ID	FC	***P***-value	Chromosome	Length (bp)
TCONS_00011823	4.85413	0.0323	Chr6	383
ENST00000425176.1	4.42477	0.0316	Chr2	317
TCONS_00008360	3.70280	0.0017	Chr4	826
XR_427694.1	3.58729	0.0315	Chr5	260
ENST00000441169.1	3.19771	0.0051	Chr3	576
TCONS_00011801	2.87082	0.0013	Chr6	543
ENST00000606596.1	2.83830	0.0013	Chr8	710
ENST00000564872.1	2.67340	0.0279	Chr18	861
TCONS_00016749	2.58434	0.0277	Chr9	548
TCONS_00026975	2.45822	0.0251	Chr19	348
NR_027146.1	2.43441	0.0001	Chr17	1549
uc.252-	2.32473	0.0222	Chr9	231
ENST00000431656.1	2.32393	0.0321	Chr13	376
ENST00000427953.1	2.32227	0.0171	Chr1	1321
TCONS_00003905	2.31021	0.0085	Chr2	628

#### Differentially expressed mRNAs

According to the microarray results, 370 mRNAs were differentially expressed (fold change ≥1.5, *P* < 0.05) between the low HDL-C disease and healthy groups, using the same criteria applied to lncRNAs. Among these, 285 mRNAs were up-regulated and 85 mRNAs were down-regulated in the disease group. The top 15 differentially expressed mRNAs are listed in Tables 4 and 5. *OLFM4* was the most significantly up-regulated mRNA (fold change: 6.86421, *P* < 0.05) and LOC102723740 was the most significantly down-regulated mRNA (fold change: 4.05710, *P* < 0.05).

**Table 4:** Top 15 up-regulated mRNAs in the low HDL-C disease group compared with those in controls

mRNA	FC	***P***-value	Chromosome
*OLFM4*	6.86421	0.0142	chr13:53626095–53,626,154
*DEFA3*	4.01720	0.0239	chr8:6873570–6,873,511
*PF4V1*	3.88238	0.0048	chr4:74720137–74,720,196
*MMP8*	3.80859	0.0493	chr11:102583793–102,583,734
*LTF*	3.45179	0.0340	chr3:46479489–46,477,720
*HBA2*	3.32376	0.0103	chr16:222889–222,948
*HBD*	3.04019	0.0123	chr11:5255317–5,255,258
*OSBP2*	2.82016	0.0355	chr22:31302848–31,302,907
*HBB*	2.78993	0.0102	chr11:5246777–5,246,718
*NFIB*	2.66136	0.0179	chr9:14088270–14,088,211
*HBG1*	2.63723	0.0469	chr11:5269593–5,269,534
*HBA2*	2.61040	0.0050	chr16:223555–223,614
*HBA2*	2.57408	0.0067	chr16:223560–223,619
*BPI*	2.54008	0.0222	chr20:36965709–36,965,768
*CAMP*	2.47498	0.0417	chr3:48266856–48,266,915

**Table 5:** Top 15 down-regulated mRNAs in the low HDL-C disease group compared with the healthy group

Gene Symbol	FC (abs)	***P***-value	Genomic Coordinates
LOC102723740	4.05710	0.0236	chr5:41284979–41,284,920
LOC101928223	3.21873	0.0310	chr4:171197767–171,197,826
LOC388456	2.58673	0.0303	chr18:926149–926,090
*JUN*	2.36467	0.0142	chr1:59246570–59,246,511
*CLEC14A*	2.30657	0.0482	chr14:38724198–38,724,139
*FAM149B1*	2.29307	0.0075	chr10:75003877–75,003,936
*MDGA1*	2.20545	0.0393	chr6:37600354–37,600,295
*RPF1*	2.14901	0.0376	chr1:84948889–84,948,948
*TLE1*	2.01499	0.0062	chr9:84214882–84,214,823
*BNC2*	1.98561	0.0147	chr9:16419008–16,418,949
*GSTO2*	1.95754	0.0039	chr10:106057346–106,057,405
LOC101928043	1.93770	0.0405	chr1:22350873–22,350,814
*RBM44*	1.85520	0.0192	chr2:238751106–238,751,165
LOC100129171	1.84261	0.0328	chr2:224854629–224,854,570
*OR3A2*	1.84140	0.0320	chr17:3181347–3,181,288

Values on the *X* and *Y* axes in the scatter plot are average normalized values for each group (log2-scaled). The lncRNAs shown above the upper red line and below the lower green line are those with expression differences of 1.5 fold change between the two groups; the red and green lines indicate the fold change threshold.

The red points in the plot represent the significantly differentially expressed lncRNAs (fold change > 1.5, *P* < 0.05), while the vertical lines correspond to 1.5-fold up- and downregulation. Horizontal line indicates *P* = 0.05.

### Bioinformatics analysis and characterization of differentially expressed LncRNAs

#### GO and KEGG pathway and disease analyses

**Fig. 6:**
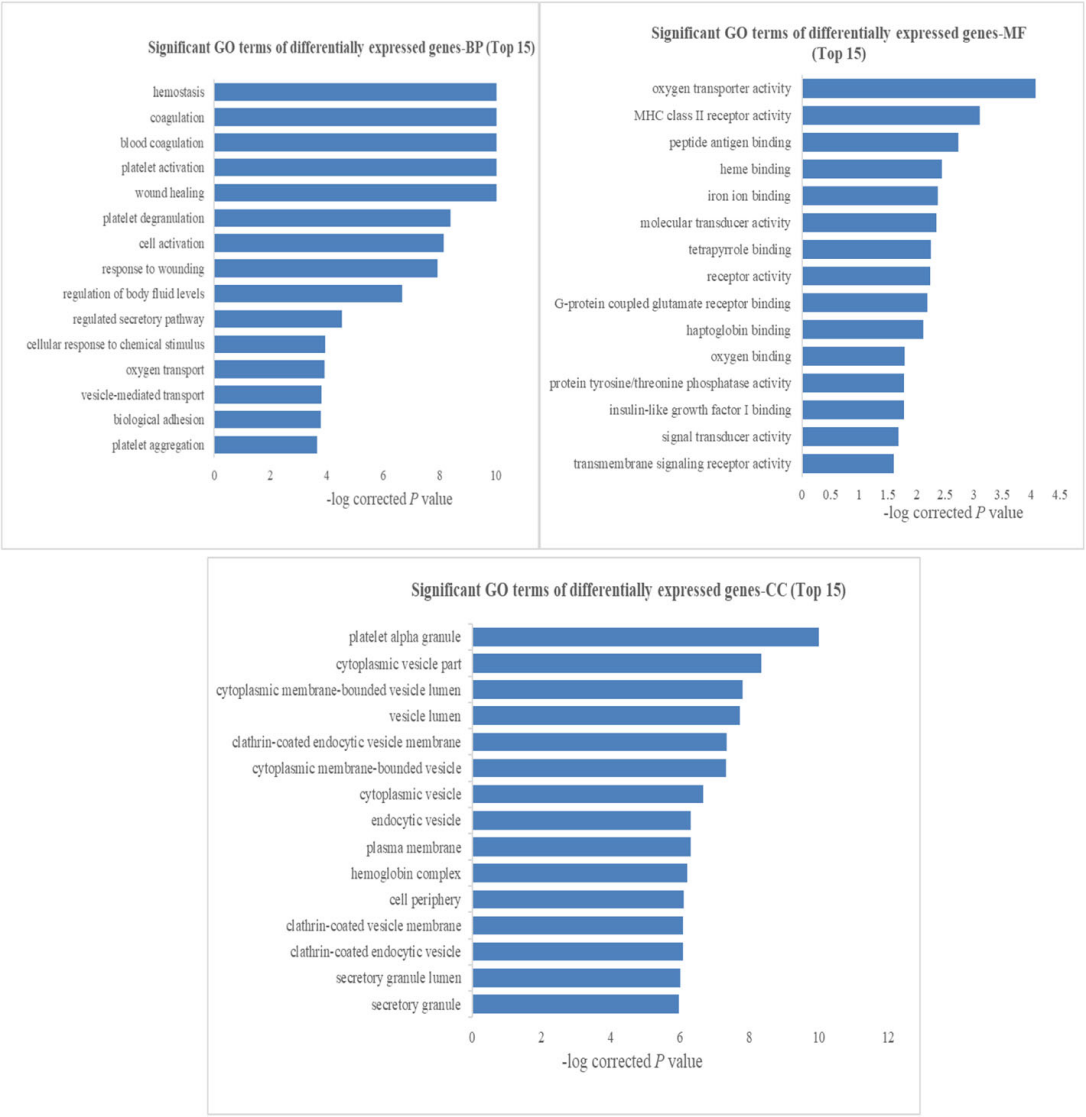
GO enrichment analysis results (top 15) for BP, MF and CC of differentially expressed mRNAs in low HDL-C disease. FDR-corrected *P* values are shown

A GO analysis was performed using Gene Ontology (www.geneontology.org), which provides three structured networks of defined terms that describe gene product attributes. The GO results showed that many differentially expressed genes were enriched in the BP, MF and CC categories, and the top 15 enriched terms are shown in Fig. 6. These results indicated that the most significantly enriched GO terms associated with differentially expressed transcripts were ‘platelet alpha granule’ (GO:0031091, ontology: cellular component; *P* = 1.98E-14), ‘wound healing’ (GO:0042060, ontology: biological process; *P* = 4.14E-14), and ‘oxygen transporter activity’ (GO:0005344, ontology: molecular function, *P* = 5.66E-07).

**Fig. 7:**
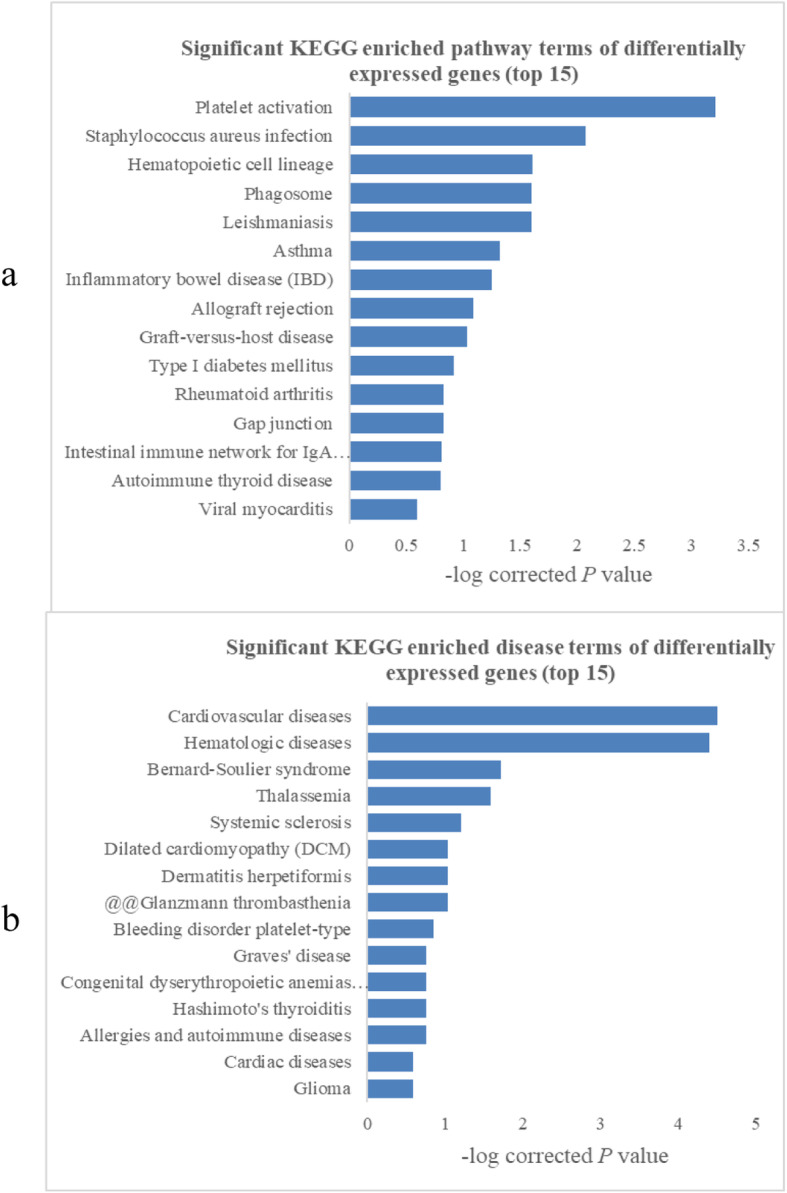
Pathway and disease analysis results (top 15) for differentially expressed mRNAs in low HDL-C disease. FDR-corrected *P*-values are shown

Additionally, a pathway analysis indicated that 23 pathways were significantly enriched among the differentially expressed transcripts (*P* < 0.05) and the top 15 pathways are shown in Fig. 7a. The most significantly enriched pathway was platelet activation (KEGG ID: hsa04611, *P* = 4.49E-06). Based on a disease enrichment analysis, we found that the most significantly enriched disease was cardiovascular disease (*P* = 3.14E-05) (Fig. 7b).

### Construction of a co-expression network

**Fig. 8:**
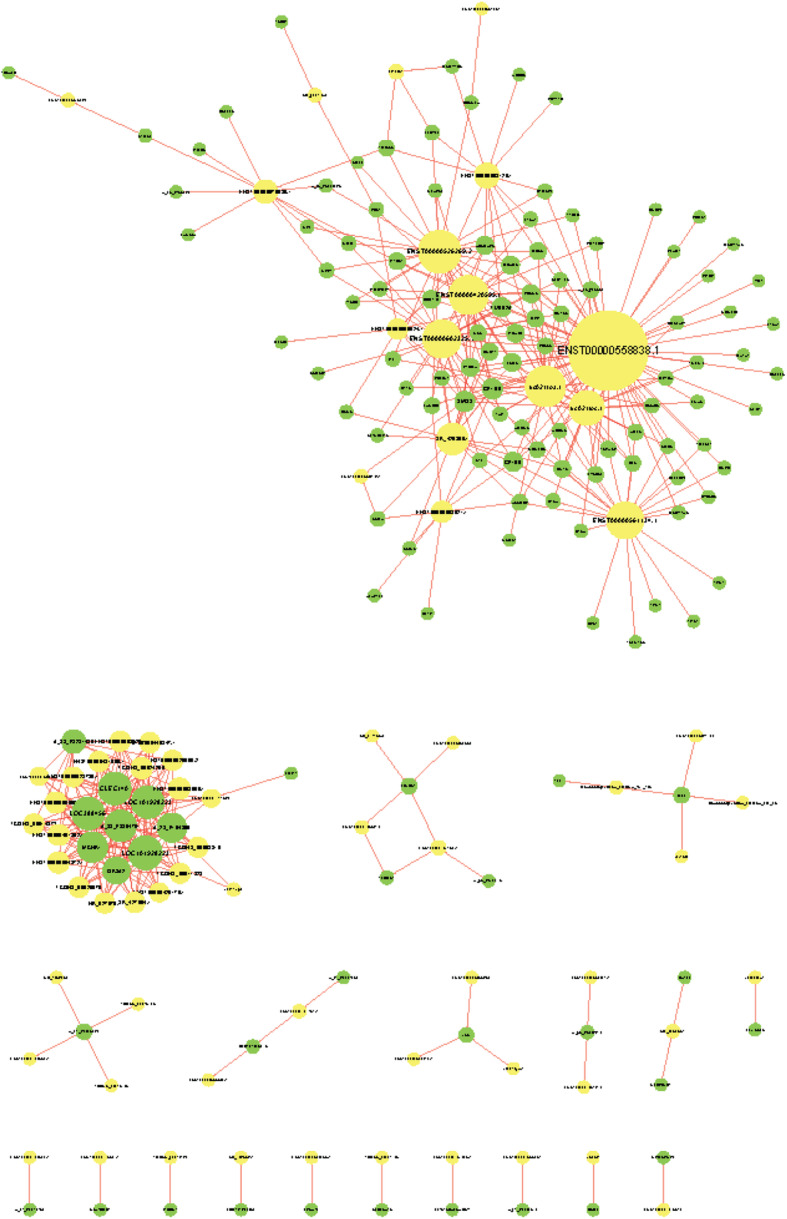
Coding–non-coding gene co-expression network. Yellow nodes represent lncRNAs, while green nodes represent mRNAs

**Fig. 9:**
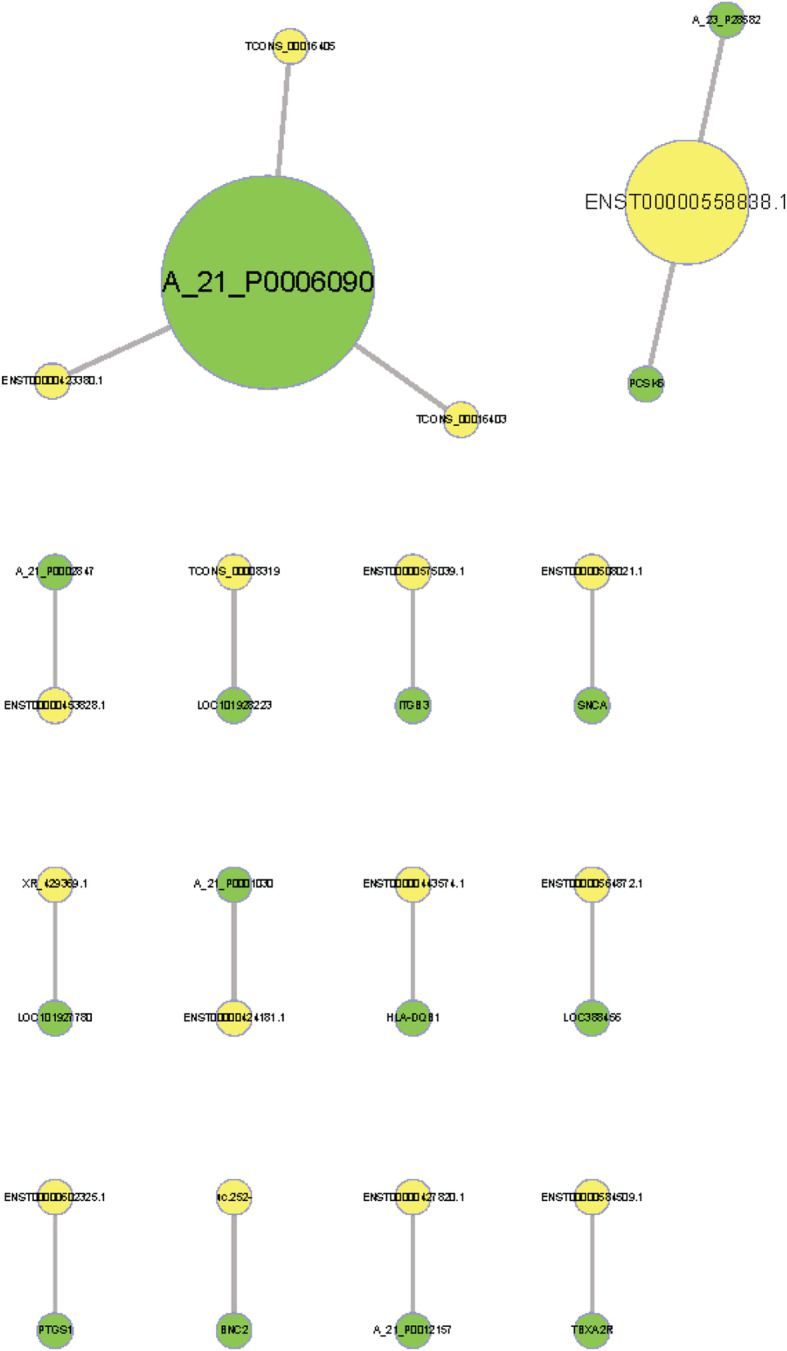
Prediction results for potential target genes of lncRNAs. Yellow nodes represent lncRNAs, while green nodes represent mRNAs

A co-expression network was constructed to explore the associations between lncRNAs and potential target mRNAs with differential expression in low HDL-C disease. Using PCC ≥ 0.90 and *P* < 0.05 as thresholds, 467 pairs of co-expressed lncRNAs and mRNAs were identified (Figs. 8 and 9).

### Long non-coding RNA target prediction

Target genes were identified for ten lncRNAs (Table 6). The lncRNA AP001033.3–201 (ENST00000584509.1) may contribute to the trans-regulation of the protein-coding gene thromboxane A2 receptor *(TBXA2R)* and the lncRNA AC068234.2–202 (ENST00000575039.1) may act a cis-regulator of the protein-coding gene integrin subunit beta 3 *(ITGB3)*. Both target genes are involved in the platelet activation pathway and cardiovascular disease.

**Table 6:** Potential target gene prediction for differentially expressed lncRNAs

lncRNA	mRNA	Correlation	***P***-value	lncRNA	mRNA
XR_429369.1	LOC101927780	0.9669	4.05165E-12	down	down
ENST00000602325.1	PTGS1	0.9436	4.45907E-10	up	up
TCONS_00008319	LOC101928223	0.9615	1.51796E-11	down	down
uc.252-	BNC2	0.9339	1.79422E-09	down	down
ENST00000508021.1	SNCA	0.9732	6.16618E-13	up	up
ENST00000564872.1	LOC388456	0.9681	2.9301E-12	down	down
ENST00000558838.1	PCSK6	0.9894	0	up	up
ENST00000575039.1	ITGB3	0.9517	1.14575E-10	up	up
ENST00000584509.1	TBXA2R	0.9206	8.85915E-09	up	up
ENST00000443574.1	HLA-DQB1	0.9378	1.05879E-09	up	up

## Discussion

Low HDL-C disease has the highest prevalence among the three types of dyslipidemia [18, 19] in the Xinjiang Kazakh population. Epidemiological studies have shown that HDL-C levels play a crucial role in the development of atherosclerosis and CAD [20], and low HDL-C disease has been identified as an independent traditional risk factor for CAD [21, 22]. Research has mainly focused on the protein-coding genes associated with low HDL-C disease [5, 7, 8, 23, 24], and little is known about the underlying genetic mechanisms, including the roles of lncRNAs, which are closely involved in various important biological processes. Researchers have shown that lncRNAs are involved in the regulation of HDL metabolism [25]; HDL in patients with CAD and hypercholesterolemia could cause the abnormal expression of lncRNAs in vascular endothelial cells and have adverse effects on vascular function. A recent microarray analysis has demonstrated that the *APOA1* gene cluster is regulated by the lncRNA *APOA1-AS* [26], which encodes APOA1, a major protein component of HDL in the plasma. Moreover, the lncRNA known as lncHR1 (HCV regulated 1) decreases lipid metabolism by repressing *SREBP-1c* gene expression [13]. However, the expression profiles of lncRNAs in low HDL-C disease have not been reported to date.

LncRNAs may function by altering the expression of protein-coding genes [27].

They are key regulators of gene expression, and dysregulated expression levels of certain lncRNAs are associated with a variety of diseases, including diseases arising from defects in lipid metabolism [12, 28, 29]. Generally, cholesterol biosynthesis in the liver is important for the subsequent synthesis of lipids and lipoproteins and for the correct functioning of lipid metabolism [30, 31]. Aberrant expression of lncRNAs may induce specific mRNAs to alter HDL-C levels, which would ultimately lead to low HDL-C disease.

In this study, genome-wide lncRNA and mRNA expression profiles were compared between individuals with low HDL-C disease and healthy individuals using a microarray approach and potential functions of differentially expressed loci were evaluated by GO and KEGG pathway analyses. A large number of differentially expressed lncRNA and mRNAs between low HDL-C disease and controls were identified. GO and KEGG pathway analyses showed that the differentially expressed genes were involved in a variety of cellular processes, cellular components, and molecular functions.

To elucidate the molecular mechanisms by which lncRNAs contribute to low HDL-C disease, a CNC network was further constructed by combining aberrantly expressed lncRNAs and mRNAs on the basis of lncRNA levels having an effect on the expression of flanking protein-coding genes [32]. The results of the current study indicated that multiple lncRNAs were clearly associated with mRNAs and the co-expression network could provide a strong foundation for predicting the functions of lncRNAs.

Additionally, a pathway analysis identified 23 pathways related to low HDL-C disease. Among these pathways, platelet activation (KEGG ID: hsa04611) involved 14 differentially expression genes and was the most significantly enriched pathway.

A disease enrichment analysis showed that 370 differentially expressed mRNAs were associated with many diseases, such as CAD, hematologic diseases, Bernard-Soulier syndrome, thalassemia, systemic sclerosis, and dilated cardiomyopathy; the greatest enrichment was observed for CAD.

Based on genomic annotation, enrichment, co-expression, and target gene prediction analyses, 10 lncRNAs with target genes were identified. Based on combined GO, KEGG pathway, and disease enrichment analyses, the lncRNAs AC068234.2–202 and AP001033.3–201 were co-expressed with the target genes *ITGB3* and *TBXA2R*.

Finally, the study results indicated that *ITGB3* and *TBXA2R* were both differentially expressed between the two groups and were both related to the platelet activation pathway and cardiovascular disease in a GO enrichment analysis. They were also co-expressed with the lncRNAs AC068234.2–202 and AP001033.3–201 in CNC networks and were identified as potential target genes of the lncRNAs AC068234.2–202 and AP001033.3–201.

The lncRNA AP001033.3–201 was a novel transcript located on chromosome 18:9,310,522–9,334,445 reverse strand. The transcript had 3 exons with 5282 reported variant alleles and mapped to 93 oligo probes. The transcript was a product of the gene AP001033.3. The lncRNA AC068234.2–202 was also a novel transcript, antisense to *ITGB3*, located on chromosome 17:47,303,474–47,323,613 reverse strand. The transcript had 3 exons, associated with 4518 variant alleles and maps to 115 oligo probes, and the transcript was a product gene AC068234.2.

Based on a literature review, *ITGB3* is an important member of the integrin family of adhesion molecules. It is located on chromosome 17:47,253,827–47,313,743 forward strand. It is associated with thrombus formation and platelet aggregation [33], abnormal platelet activation and abnormal thrombosis [34], and inflammatory responses and atherosclerosis. *TBXA2R,* located on chromosome 19:3,594,507–3,606,875 reverse strand, may affect platelet function and venous thrombosis [35], platelet aggregation, and the process of atherosclerosis. Usually, *TBXA2R* is widely distributed in the cardiovascular system. However, the distribution of *TBXA2R* is altered in various cardiovascular diseases and is involved in pathophysiological processes. Platelet activation plays an important physiological role in inflammation, and extensive clinical and experimental evidence supports the importance of inflammatory processes in both the initiation and development of dyslipidemia [36, 37].

Based on the above analysis, the results of this study suggest that the lncRNA AC068234.2–202 is a cis-acting regulator of *ITGB3* and the lncRNA AP001033.3–201 is a trans-acting regulator of *TBXA2R,* contributing to the induction of platelet activation and subsequently to the pathogenesis of CAD. These results provide a foundation for further investigations of lncRNA functions, signaling pathways, and roles in low HDL-C disease.

To conclude, the expression profiles of lncRNAs and mRNAs in low HDL-C disease were determined using a microarray approach, and 381 lncRNAs and 370 mRNAs were identified. An lncRNA–mRNA co-expression network and predicted network were constructed and combined with the results of GO, KEGG pathway, and disease enrichment analyses. Results indicated that the lncRNA AP001033.3–201 is a trans-regulatory element for the protein-coding gene *TBXA2R* and the lncRNA AC068234.2–202 is a cis-regulatory element for the protein-coding gene *ITGB3*. Both of these lncRNAs were involved in platelet activation and CAD. Based on these results and microarray data, the results suggest that platelet activation plays an important role in the occurrence and development of low HDL-C disease.

Moreover, Zhong et al. reported that both high and low HDL-C levels (ranging from 22 mg/dL to 97 mg/dL) were associated with an increased risk of mortality from CAD [38]. The lncRNAs identified in this study provide insights into the potential molecular mechanisms underlying the effects of low HDL-C levels. However, further research is needed to confirm the functions of the candidate differentially expressed lncRNAs and their potential regulatory relationships in low HDL-C disease.

### Study strengths and limitations

A large number of differentially expressed lncRNAs and mRNAs in low HDL-C disease were successfully screened in a Kazak population and potential target genes were predicted by microarray and bioinformatic analyses. However, the study had certain limitations. First, it was based on the results of bioinformatics analyses, and further biological experiments, such as qPCR on an independent cohort, are needed to verify the findings. Second, the cross-sectional study design could not reveal whether changes in lncRNA and mRNA expression were primary or secondary changes in the disease. Finally, it is important to acknowledge that the small study cohort might contribute to a reduced statistical power, to some extent.

## Conclusions

In this study, lncRNA and mRNA expression profiles in low HDL-C disease were screened in a Kazak population, and a large number of lncRNAs and mRNAs were found to be differentially expressed. GO, KEGG pathway, and disease analyses suggested that the lncRNAs AC068234.2–202 and AP001033.3–201 may regulate the genes *ITGB3* and *TBXA2R,* respectively. These loci are involved in platelet activation and the pathogenesis of CAD. This microarray analysis clarified the expression patterns of lncRNAs in low HDL-C disease and laid the foundation for future functional and mechanistic studies of lncRNAs associated with low HDL-C disease.

Furthermore, key candidate genes associated with low HDL-C disease were identified, providing insights into the underlying genetic basis as well as a basis for follow-up research, including functional analyses of lncRNAs and studies of the roles of signaling pathways in the HDL-C disease in the Kazakh population in Xinjiang. In addition, the results of this study improve our understanding of the etiology of both low HDL-C and CAD as well as the relationship between the diseases.

## Data Availability

The datasets used and/or analyzed during the current study are available from the corresponding author on reasonable request.

## Abbreviations

HDL-C: High-density lipoprotein cholesterol
*CETP*: Cholesterol ester transfer protein
*ABCA1*: ATP-binding cassette transporter A1
*APOA-1*: Apolipoprotein A-1
*lncRNAs*: Long non-coding RNAs
CAD: Coronary artery disease
*ITGB3*: Integrin beta-3
*TBXA2R*: Thromboxane A2 receptor
